# Hydroxide films on mica form charge-stabilized microphases that circumvent nucleation barriers

**DOI:** 10.1126/sciadv.abn7087

**Published:** 2022-09-02

**Authors:** Benjamin A. Legg, Kislon Voïtchovsky, James J. De Yoreo

**Affiliations:** ^1^ Physical Sciences Division, Pacific Northwest National Laboratory, Richland, WA 99354, USA.; ^2^ Department of Materials Science and Engineering, University of Washington, Seattle, WA 98195, USA.; ^3^ Department of Physics, Durham University, Durham DH1 3LE, UK.

## Abstract

Crystal nucleation is facilitated by transient, nanoscale fluctuations that are extraordinarily difficult to observe. Here, we use high-speed atomic force microscopy to directly observe the growth of an aluminum hydroxide film from an aqueous solution and characterize the dynamically fluctuating nanostructures that precede its formation. Nanoscale cluster distributions and fluctuation dynamics show many similarities to the predictions of classical nucleation theory, but the cluster energy landscape deviates from classical expectations. Kinetic Monte Carlo simulations show that these deviations can arise from electrostatic interactions between the clusters and the underlying substrate, which drive microphase separation to create a nanostructured surface phase. This phase can evolve seamlessly from a low-coverage state of fluctuating clusters into a high-coverage nanostructured network, allowing the film to grow without having to overcome classical nucleation barriers.

## Teaser

Al(OH)_3_ films grown on a mica-water interface form via nanostructured surface phases that are stabilized by electrostatic forces.

## INTRODUCTION

The nucleation and growth of minerals from solution is a critical process in materials synthesis, biomineralization, and geochemistry. Similar to most phase transformations, mineral precipitation is facilitated by statistical-mechanical fluctuations, but the exact nature of these fluctuations may vary widely between systems. The historically dominant model for understanding precipitation is classical nucleation theory (CNT), in which relevant fluctuations are assumed to be clusters of atoms or ions, commonly referred to as embryos or nucleation clusters (*1*–*4*). By making ancillary assumptions about the energy of cluster formation (e.g., the capillarity approximation) and mechanism by which clusters form (e.g., addition and subtraction of monomers units), CNT provides a working model for mineral nucleation. However, many alternative pathways of mineral precipitation have recently been proposed (*5*–*9*), which are based on different assumptions about the nature of the relevant fluctuations and the energy landscapes that govern their formation.

Ideally, one could determine the pathway of mineral precipitation by directly imaging the relevant fluctuations, but this has been extraordinarily challenging, because the structures involved are intrinsically small, rare, short lived, and difficult to detect. To date, CNT-like processes have only been imaged for a few special cases, such as metallic nanoparticles (*10*) and two-dimensional (2D) protein crystals (*11*, *12*), and these results cannot be directly extrapolated to mineral-water systems, because the materials involved have markedly different chemical-physical properties.

However, recent advances in atomic force microscopy (AFM) provide an exciting tool for approaching this problem. Modern high-speed AFM is able to resolve to image individual ions and ion clusters at mineral-water interfaces with near-atomic resolution (*13*–*16*), thus providing the resolution that is needed to directly observe the fluctuations that facilitate nucleation. By using AFM to observe nanoscale fluctuations, characterizing both their dynamics and size distributions, we can reveal the energy landscapes that control mineral precipitation and the pathways by which these energy landscapes are traversed.

Here, we apply AFM to study the formation of aluminum hydroxide films at a mica-water interface. This has recently emerged as a model system for studying mineral nucleation following previous work demonstrating that when the (001) mica surface is exposed to aqueous aluminum salt solution, it can concentrate hydrolyzed aluminum ions near the surface and promote the nucleation and growth of gibbsite-like aluminum hydroxide epitaxial films (*17*–*19*). The system is uniquely suited for AFM investigation, because the resulting 2D films are atomically flat (*20*), and even single-ion precursors can be resolved under ideal conditions (*15*).

The aluminum-mica system is important to geochemistry (e.g., aluminum cycling, mineral dissolution, and soil mechanics) (*21*) and materials synthesis (e.g., green synthesis of Al_2_O_3_ thin films) (*22*), and it allows us to explore numerous phenomena that are specific to mineral-water systems. In particular, the combination of mica and aluminum gives us the opportunity to understand how surface charging influences the energy landscape for heterogenous nucleation and growth. Unlike many model systems for which CNT has been developed and tested (such as metallic alloys), mineral-water systems often display complex surface-charging behavior due to ion adsorption and surface complexation. Surface charging has been implicated in many past studies on heterogenous mineral nucleation (*23*–*26*), but no quantitative mechanistic models exist to predict its effects. In the absence of models that can rigorously treat surface charging, most studies of heterogenous nucleation simply preserve the basic framework of CNT while making minor adjustments, such as assuming that the interface reduces the size (*27*) or effective surface tension (*2*, *26*, *28*) of the critical nuclei. However, electrostatic fields have the capability to induce much more complex effects.

In this study, we directly observe the fluctuations that precede film growth and investigate how surface charging can induce systematic deviations from the classically assumed energy landscapes to enable alternative pathways of precipitation. This is an important step toward developing next-generation models of nucleation and growth at mineral-water interfaces.

## RESULTS

### Characterizing film growth by AFM

**Fig. 1.**
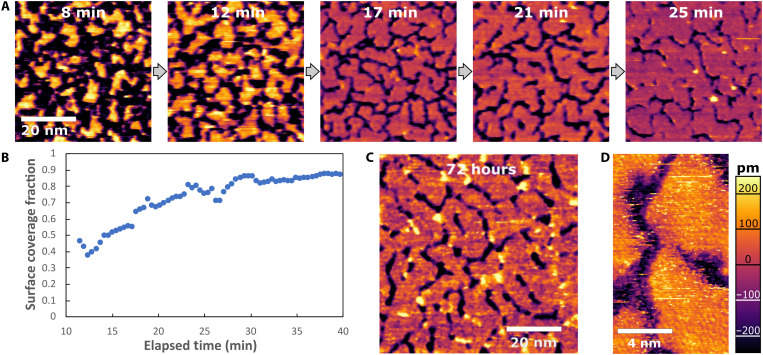
In situ AFM images of gibbsite nucleation and growth at the mica-water interface. (**A**) In situ AFM image sequence from movie S1, showing the growth and coalescence of clusters to create an extended film, with a persistent network of gaps, obtained in a pH 3.7 solution (1 mM AlCl_3_ + 0.2 mM HCl) at 65°C. (**B**) Corresponding plot of surface coverage versus elapsed time (relative to time when temperature began rising to 65°C). (**C**) Image of a film aged ex situ at the same conditions for 72 hours and then imaged in situ at room temperature. (**D**) High-resolution in situ AFM of a sample aged at similar conditions for several days, revealing atomistic details of the surface crystallinity and morphology of the gibbsite monolayer, including extensive crystalline defects. Image obtained at room temperature, after growth.

Mica surfaces exposed to aqueous aluminum chloride solutions are known to produce epitaxial gibbsite-like films when gently heated (*18*, *20*), but the pathway of film formation has not been previously observed. Here, time-resolved AFM imaging of film growth in 1 mM AlCl_3_ and 0.2 mM HCl (pH ~ 3.7) solutions at 65°C reveals the gradual formation of extended monolayer films (see Fig. 1A and movie S5). The earliest stable, high-resolution images (obtained after ~8 min of reaction time) reveal a surface that is heavily covered by dynamic, island-like clusters, and these clusters grow and coalesce to form a continuous flat film with an apparent height of 3 to 6 Å over the course of 25 min.

The growth dynamics are intriguing, in that the film appears to asymptotically approach partial coverage (see Fig. 1B). This is confirmed by imaging of films aged in an oven for several days, which retain the same pattern of partial coverage, with a persistent network of gaps (Fig. 1C). This morphological evolution points to an inconsistency with simple models of crystal growth, because we would typically expect to rapidly fill in the narrow gaps between islands (especially where the island perimeters display a high negative curvature). Although we consider the possibility that these gaps may result from strain at antiphase boundaries where domains of different epitaxial registrations meet, this cannot fully explain the observations, because many gaps intrude into islands that are otherwise contiguous. Moreover, the gaps seem larger (often several nanometers) than would be needed to relieve strain, and high-resolution imaging of aged films shows that the islands have poor crystallinity, with large numbers of defects such as dislocations that should be capable of accommodating any strain between different domains of registration (Fig. 1D and fig. S8). Thus, we must begin to consider alternative explanations, such as the possibility that electrostatic forces favor the formation of nanostructures that intermix positively charged aluminum hydroxide–coated regions with negatively charged regions of bare mica.

**Fig. 2.**
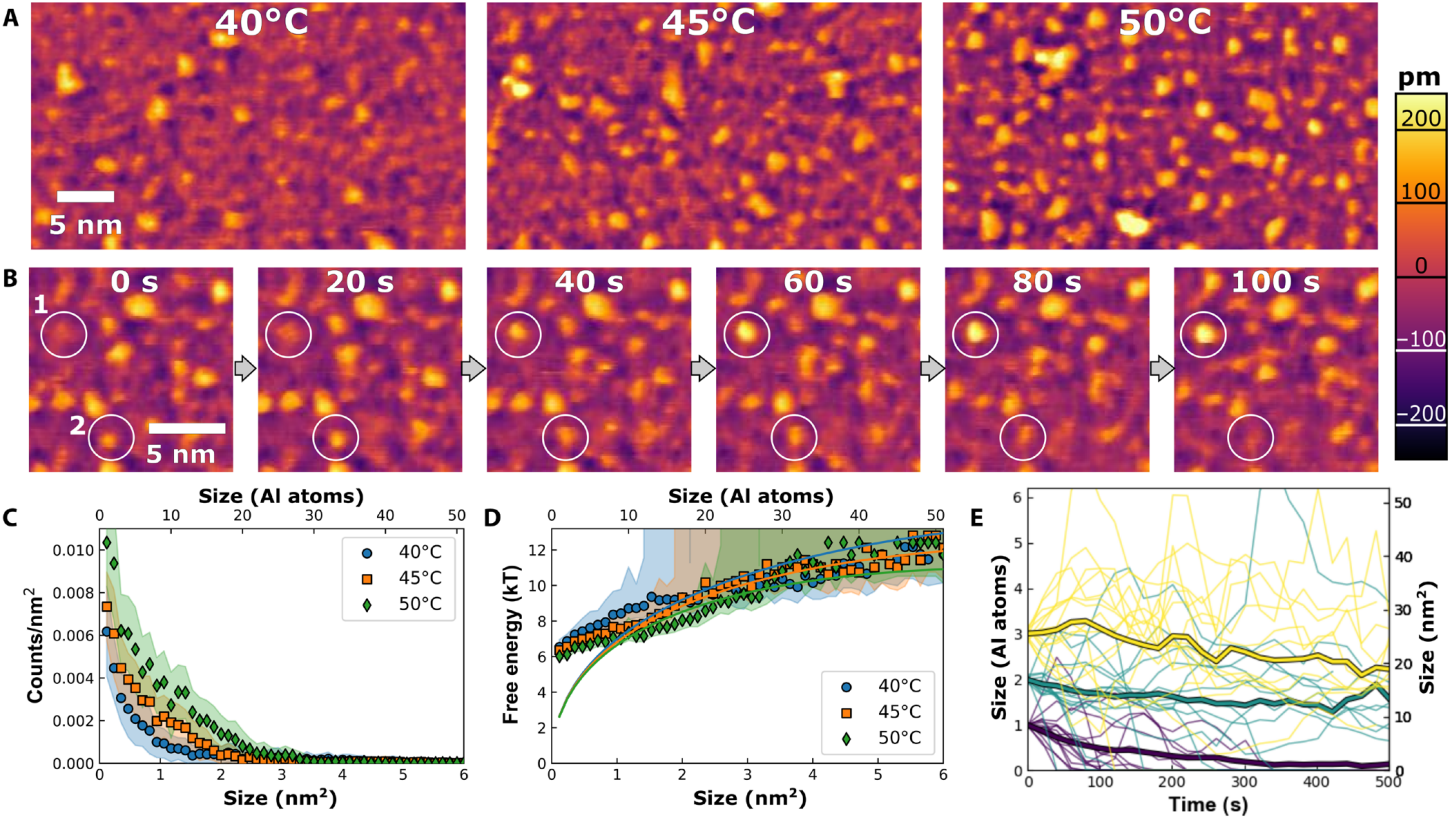
Cluster size distributions and size fluctuations. (**A**) Still frames from movies S1 to S3, showing precursors to film formation, and the emergence of nanometer-scale clusters as the temperature is increased from 40° to 50°C in a pH 3.7 solution (1 mM AlCl_3_ + 0.2 mM HCl). (**B**) Image sequence from movie S3 (50°C), showing fluctuations in cluster size over time. Highlighted region 1 shows a cluster appearing and growing, while region 2 shows a cluster changing shape and disappearing. (**C**) Cluster population distributions, measured at 40°, 45°, and 50°C. Color bands show ±1 SD from the mean. (**D**) Calculated cluster energy landscapes obtained by applying Eq. 1 to the population distributions in (A). Lines show attempts to fit the energy landscape using the classical capillarity approximation. (**E**) Trajectories of ensemble-average size for initially monodisperse ensembles of clusters (heavy lines, outlined in black), along with size trajectories for selected individual clusters from those ensembles. Ensembles were extracted from the 50°C dataset. Ensembles were prepared with initial sizes near 3.0 nm^2^ (yellow), 2.0 nm^2^ (cyan), or 1.0 nm^2^ (purple). Although individual clusters may either grow or shrink, the average behavior shows a clear tendency to shrink.

We gain additional insights by performing time-resolved experiments at temperatures, *T*, between 40° and 55°C, just below the threshold needed to grow a continuous film. We note that the precipitation of aluminum hydroxide is driven by the formation of hydroxyl bridges. Because of the associated liberation of solvating waters and protons, this is an endothermic process, and the driving force for precipitation, thus, increases with *T*. Drawing insights from classical theories, we expect structural fluctuations to grow and increase in intensity as the temperature is increased toward the threshold for film growth. Here, we observe large populations of isolated clusters (Fig. 2A) that fluctuate in both size and shape, with individual clusters appearing and disappearing over the course of minutes (Fig. 2B and movies S1 to S4). The clusters are relatively flat, with apparent heights on the order of angstroms, which is consistent with the expectation that they are fragments of a gibbsite-like monolayer. The size, location, and number of clusters are determined by height threshold segmentation (see Materials and Methods and figs. S2 and S3 for image processing details). We find that as *T* is increased toward the threshold for film growth, both the surface coverage and the total number of clusters increase (Fig. 2A). Coverages range from roughly 5% at 40°C to 10% at 50°C, but no extended films form until *T* is raised above 55°C.

These time-resolved AFM images of fluctuating clusters provide a notable confirmation of thermal fluctuations before phase transformation, and they display several properties that are reminiscent of CNT. First, the fluctuations can be modeled as localized clusters that fluctuate size, as postulated by CNT. Moreover, the cluster size distribution decays exponentially with size (Fig. 2C), which is reminiscent of CNT-based models, for which smaller subcritical clusters are expected to be common, while larger clusters will be rare because of their increasingly high energies of formation (*4*, *29*).

One advantage to working at temperatures below the threshold for film growth is that the surface coverage is steady with time, enabling us to apply equilibrium thermodynamics to determine free energies of cluster formation via the Boltzmann relation, as shown in Eq. 1

$$∆G_{n}=-k_{B}T\text{ln}N_{n}/N_{0}$$ (1)

where ∆*G_n_* is the free energy of formation for a cluster of *n* aluminum atoms, *N_n_* is the concentration of clusters with that size, and *N*_0_ is the density of sites where clusters can form. As expected, we calculate ∆*G_n_* values that increase with size (Fig. 2D).

The obtained energy landscapes can then be compared with common geometric scaling laws, such as the capillarity approximation. For 2D islands, the capillarity approximation treats ∆*G_n_* as the sum of an area-dependent and edge-dependent term as follows

$$∆G_{n}=n\Delta \mu_{s}+\tau \sqrt{a_{o}4\pi n}$$ (2)

where Δμ_s_ reflects the chemical driving force to nucleate a film (we use the subscript s to denote that this value is specific to the surface film and may be offset relative to the value for bulk gibbsite, Δμ_b_), τ is the cluster edge tension, and *a*_o_ is the area per aluminum atom in the final film (0.1177 nm^2^). However, this expression does a poor job of fitting the energy landscape. It struggles to capture the energy of the smallest clusters, and our best-fit attempts require very small values of τ = 2.3 *kT*/nm (or roughly 0.01 nJ/m) to reproduce the gentle slope of the energy landscapes, which is unexpectedly small, because estimated edge tensions for bulk gibbsite sheets should be on the order of ~30 nJ/m (see section S7). Furthermore, once τ is assigned, the fits constrain us to a very narrow range of Δμ_s_ (−0.156 *kT* at 40°C to −0.160 *kT* at 50°C), which is also unexpectedly small, because thermochemical models (*30*) predict that Δμ_b_ should change by roughly 1 *kT* (a factor 250 times greater) over the same temperature range. It is not unexpected that Δμ_s_ differs from Δμ_b_, because the film energetics are expected to be offset from bulk by the addition of gibbsite-mica and gibbsite-water surface energy terms that also scale with cluster area. More unexpected is the fact that Δμ_s_ remains so close to zero across a range of temperatures where Δμ_b_ varies markedly. The offsetting surface energies are not expected to have the same temperature dependence as Δμ_b_, so the insensitivity of Δμ_s_ to changes in temperature points to a more complex, self-regulating phenomenon. Thus, we find that the energy landscape for generating gibbsite surface clusters is not simply related to the energetic parameters for forming bulk gibbsite, as would be assumed in most models of heterogenous nucleation. The cluster edge tensions are markedly reduced relative to bulk gibbsite, and the cluster populations are not nearly as sensitive to changes in saturation as would be expected.

Despite this failure of the capillarity approximation to predict cluster energies, the cluster fluctuations still display many of the behaviors predicted by CNT, once the modified energy landscape is accounted for. The clusters fluctuate in size incrementally, with large jumps being rare. This is consistent with the model envisioned in CNT, where most changes involve adding or subtracting monomer units (although dimers, trimers, or other small species may also contribute). Furthermore, relatively little of the size flux (less than 15%) involves cluster splitting or merging. In addition, although we occasionally observe large particles landing on the surface (which might indicate particle-mediated growth), these can be distinguished from surface-formed clusters on the basis of their greater height and mobility, and they are also rare (comprising less than ~10% of the observed features).

We further quantify the cluster fluctuations by identifying ensembles of like-sized clusters (regardless of when they appear during the experiment) and by tracking their subsequent evolution to generate size trajectories (Fig. 2E and fig. S4). Individual clusters show strong stochastic behavior, with some clusters growing and others shrinking, but the ensemble average size consistently shrinks, indicating that the energy landscape favors dissolution (i.e., ∂∆*G_i_*/∂*i* is positive). Thus, all of the clusters would seem to be classified as “subcritical” in the language of CNT. By modeling the ensemble dynamics as a biased Markovian random walk (details in sections S4 to S6), we estimate that the slope of the energy landscape, $\frac{\partial \text{∆}G_{i}}{\partial i}$, is on the order of 0.01 to 0.1 *kT* for each the plotted ensembles (see section S6 and fig. S7). Although the data are too noisy to extract the dependence of the slope on temperature or size, the fluctuation analysis still reveals a gently sloped energy landscape that is in reasonable agreement with the values derived from cluster populations (slopes of ~0.1 *kT*), as seen in Fig. 2D. Qualitatively similar trajectories can be generated by kinetic Monte Carlo (KMC) simulations of cluster fluctuations on classical energy landscapes (fig. S5), so long as the energy landscapes involved have similarly gentle slopes, but simulations with steeper energy landscapes produce less stochastic and more deterministic behavior. However, simulations performed with CNT-based energy landscapes predict runaway growth when clusters exceed a critical size, and this is not observed experimentally below 55°C. Instead, our AFM studies show that clusters even as large as 3 nm^2^ have a clear tendency to shrink, and we occasionally observe clusters as large as 6 nm^2^ that do not show any special tendency to grow. This indicates that a critical size (if one even exists) must be even larger.

In summary, observations of clusters before the onset of film precipitation show an exponentially decaying population of unstable clusters, which appear to fluctuate in size via classical mechanisms but whose energy landscapes do not obey the standard classical scaling laws. Instead, it appears that additional forces are present at the interface that act to reduce the effective cluster edge tensions and that also resist large changes in surface coverage, such that large changes in the chemical potential driving force to nucleate bulk gibbsite do not produce correspondingly large changes in the populations of surface clusters.

### Monte Carlo simulations

**Fig. 3.**
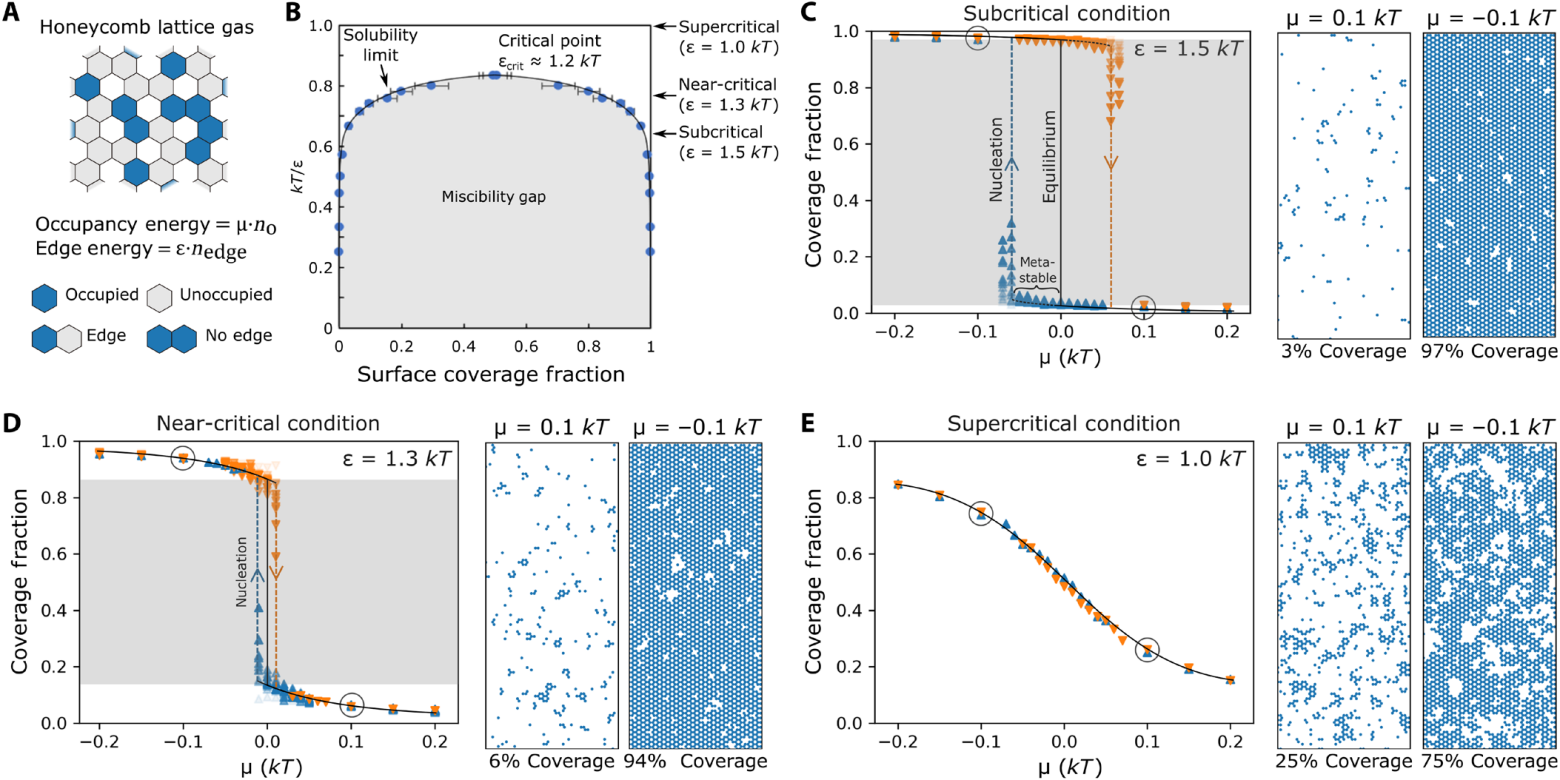
Monte Carlo simulations of a noncharging 2D lattice gas. (**A**) Schematic of the 2D honeycomb lattice simulation construction. (**B**) Corresponding phase diagram determined through Monte Carlo simulations, showing miscibility gap for ε > 1.2 *kT*. (**C** to **E**) Summaries of Monte Carlo simulations for three values of ε. Right: Images of each subfigure show equilibrium configurations at μ = 0.1 *kT* and μ = −0.1 *kT*. Left: Plots of each subfigure show surface coverage versus μ. Blue triangle (▲) symbols represent simulations initialized from zero coverage; orange inverted triangle (▼) symbols represent simulations initialized with full coverage. Circled points correspond to configurations plotted on right-hand side. Black curves indicate the equilibrium coverage, and gray bands denote the unstable coverage range within the miscibility gap. Dashed colored lines (with trailing symbols) represent potential trajectories across the coverage gap. (C) A subcritical system (ε = 1.5 *kT*), which only allows very high (>95%) or very low (<5%) coverages. Conditions with |μ| < 0.05 may persist within the gap during the simulation, but rapid nucleation is observed when |μ| exceeds 0.07 *kT*. (D) A near-critical system (ε = 1.3 *kT*), in which higher stable coverages (~15%) can be achieved but very small supersaturations with |μ| < 0.01 *kT* can induce nucleation. (E) A supercritical system (ε = 1 *kT*), where the coverage becomes a smoothly varying function of μ and all coverages can be attained, including states with high populations of fluctuating clusters.

To gain deeper insights into the mechanisms at play, we conducted Monte Carlo lattice gas simulations. Lattice gas simulations have been previously proven useful for understanding nucleation kinetics (*31*), heterogenous nucleation (*32*), deviations from capillarity (*29*, *33*), and nonclassical nucleation pathways (*7*). Lattice gas simulations are ideally suited for this system, because the pseudo-hexagonal mica surface provides a natural lattice of sites, upon which aluminum ions can adsorb, desorb, and form clusters with neighbors (Fig. 3A).

We first simulated nucleation with a 2D honeycomb lattice gas model, using a simple energy landscape defined by the cluster edge energy, ε, and chemical potential, μ (details in the Supplementary Materials), which is analogous to the 2D Ising model (*29*). The edge energy in a lattice gas model can be directly mapped to the nearest-neighbor bond energy, so that stronger bonds between neighboring surface aluminums is reflected as a higher edge energy. In this model, a miscibility gap emerges when ε exceeds a critical value of ε_crit_ ≈ 1.2 *kT* (*34*). These systems with ε > ε_crit_ are termed subcritical. In these systems, surface coverages within the miscibility gap cannot be sustained without nucleating a secondary phase (Fig. 3B). For example, when ε = 1.5 *kT*, the equilibrium coverage jumps abruptly from ~4 to ~96% when μ passes from positive to negative (Fig. 3C). Intermediate coverages can exist temporarily as metastable states, but they are very sensitive to nucleation, and our simulations show that values of μ < −0.07 *kT* will drive rapid nucleation of a film (in this simple case, μ is directly related to the driving force for film nucleation, Δμ_s_ in Eq. 2). The maximum sustainable coverage can be increased if ε is reduced toward the critical point. For example, equilibrium coverages can exceed 10% when ε = 1.3 *kT* (Fig. 3D), but then nucleation can be triggered with very small driving forces (Fig. 3D). When ε is further reduced below ε_crit_, the miscibility gap disappears and the equilibrium coverage becomes a smoothly varying function of μ (Fig. 3D), with no discontinuity. The coverage thus follows a simple ion adsorption isotherm, rather than showing precipitation of a distinct phase via nucleation. With no phase boundary, these systems can be described as supercritical. At this point, the mapping between Δμ_s_ and μ is lost, because Δμ_s_ is not defined in the absence of a phase boundary.

When comparing this model with the experiments, the high, stable cluster coverages observed by AFM (where up to 10% of the surface is covered with clusters) can only be rationalized if the system is either near-critical or supercritical. This would also explain the low edge tensions required to fit the cluster energy landscapes, because near-critical systems have intrinsically low edge tensions (*29*, *34*). However, a supercritical or near-critical behavior of the gibbsite film on mica is somewhat unexpected, because bulk gibbsite displays low solubility in bulk solution (~0.02 mM Al^3+^ at pH 4), which is the signature of a highly subcritical system with strong interactions between ions. A transition from subcritical behavior in bulk to supercritical behavior on the surface could be rationalized if the bonds between neighboring aluminum ions were disrupted at the surface. However, this would still be unexpected because past work has assumed that these gibbsite films form via a process of nucleation and growth, which implies the existence of a phase boundary. Moreover, the experimental observation of clusters with gibbsite-like structure suggests that at least moderate short-range interactions between ions are still present. An alternative explanation is that longer-range interactions between non-neighboring ions cause deviations from the simple lattice-gas model.

We propose that the experimental behavior can be understood by considering how long-range electrostatic forces from surface charging modify the cluster energy landscape and stabilize intermediate film coverages. This is a reasonable expectation because surface charging is well-known to affect aluminum-mica interactions. Surface potentials of tens to hundreds of millivolts have been observed for mica immersed in AlCl_3_ solutions (*15*), and these potentials bias ion adsorption (*35*). If surface charging modifies the adsorption energy for small ions, then it must naturally affect the energy of formation for nanoscale clusters too. Past studies have suggested that surface charging is an important driver for gibbsite film stability on mica (*36*), because 2D film growth is favored in pH ranges where mica and gibbsite display opposite charge. However, while qualitative correlations between surface charge and heterogenous mineral nucleation rates have been noted in past literature (*26*, *28*), there are still no rigorous models for predicting exactly how surface charging modifies the energy landscape for nucleation and growth. This knowledge gap is significant, considering the ubiquity of surface charging in real-life systems.

**Fig. 4.**
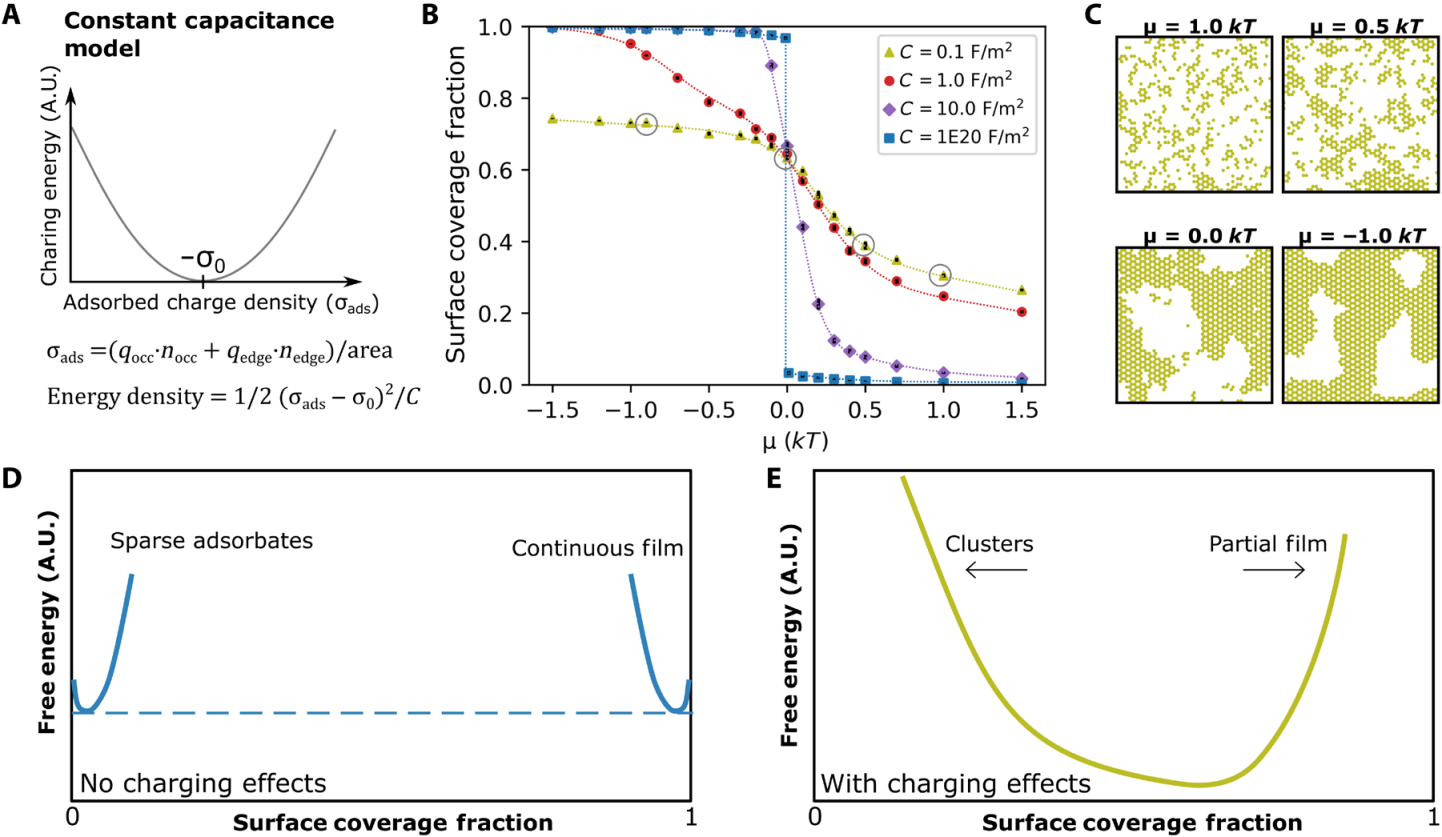
Impact of charging on KMC-predicted surface coverages. (**A**) Schematic of constant capacitance model, showing the energy penalty that emerges when net surface charge deviates from zero. (**B**) Coverage versus chemical potential diagram for KMC models that include a constant-capacitance surface charging term, showing the evolution from a sharp step function when surface capacitances are high to a smoothly varying sigmodal function when surface capacitances are low. Circled points designate the conditions for which coverages are shown in (C), and black error bars denote fluctuations in coverage (±1 SD). (**C**) Late-stage surface configurations from simulations with *C* = 0.1 F/m^2^, showing clusters for μ > 0 and continuous partial films when μ < 0. (**D**) Cartoon of a free energy landscape for the simulations without charging effects. The abrupt change in coverage seen in (B) corresponds to the presence of a dashed tie line in this subfigure, which is traversed via a traditional first-order phase transformation between a sparse adsorbate phase and a continuous film. (**E**) Cartoon of a free energy landscape for a system with substantial charging effects. The free energy landscape is now represented by a single free energy curve, so that the system transitions smoothly between a low-coverage cluster state and a partial continuous film state. A.U., arbitrary units.

To test the above hypothesis and understand the influences of surface charging on nucleation, we incorporated charging into our lattice gas model via a hybrid approach where we treat nearest-neighbor interactions (such as those emerging from hydroxyl bridges) using the traditional lattice-gas methods but incorporate long-range electrostatic interactions in a mean-field manner by adapting the constant capacitance model of ion adsorption (see Fig. 4A) (*35*). In the constant capacitance model, the surface is treated as a plane capacitor that has minimum electrostatic energy when the total bound surface charge, σ_bound_, is zero. (Although, note that in real systems, the electrostatic energy may actually be minimized at non-neutral conditions because of the organization of charge into multiple layers). In our model, σ_bound_ is determined by summing the structural charge density of the mica surface, σ_0_, which is negative, and by summing charge density due to aluminum-hydroxide adsorbates, σ_ads_, which is positive. In a standard constant capacitance model, adsorbed charge is determined simply by counting the specifically adsorbed ions, but in our model, each cluster was assumed to impart a quantity of positive charge that depends on the cluster’s area and edge length (details in Materials and Methods). Adsorbed charge is translated into charge density by normalizing by surface area. Edge charge is the primary source of charge for bulk gibbsite particles, but we included an area-scaling term because there is evidence that the gibbsite basal plane can carry substantial charge when films are defective (*37*, *38*), and our AFM images shows that the gibbsite films are highly defective even after extended aging (see fig. S8). The modeled electrostatic energy increases parabolically as σ_ads_ + σ_0_ deviates from zero, and its magnitude is inversely related to the surface capacitance. Here, we explore surface capacitance values that span from *C* = 1 × 10^20^ F/m^2^ (a physically unlikely scenario, chosen to reflect the limit of no charging effects) to *C* = 0.1 F/m^2^ (corresponding to substantial but physically reasonable charging effects). Using these values, we calculated a range of electrostatically corrected KMC coverage curves (Fig. 4B). These show that the equilibrium coverage changes from a sharp step function to a smoother and more sigmoidal shape as *C* is decreased.

The precise impact of surface charging depends on whether the surface is undercharged or overcharged relative to the ideal charge state. If undercharged (typically when μ > 0), then electrostatic forces drive more aluminum cations to the surface (Fig. 4B) and also favor the dissociation of aluminum into smaller clusters that can carry more positive edge charge. Surface charging therefore tends to favor high populations of small clusters when μ > 0 (see Fig. 4C, μ = 1.0), thus creating a population of small fluctuating clusters that is reminiscent of the AFM observations from 40° to 50°C. However, the charging forces have opposite effects when the surface is overcharged (typically μ < 0). Then, the electrostatic forces inhibit adsorption of more cations and limit the film to partial coverage (Fig. 4B), and they cause the film to coarsen because they favor the elimination of excess edge charge. The resulting morphology (see Fig. 4C, μ = −1.0) is reminiscent of the AFM observations above 65°C, in which an extended gibbsite film is interspersed with a network of persistent gaps. Surface electrostatics can thus rationalize both high coverages of clusters at low saturations and the persistence of a partial film at high saturations.

The effects can be visualized as changes to the underlying free energy landscape, as demonstrated by the cartoons in Fig. 4 (D and E). In the absence of charging effects, film nucleation involves an abrupt transition from a sparse adsorbate phase to a continuous film phase via a first-order transformation. In the corresponding free energy landscape, we thus see two distinct energy curves, joined by a tie line (Fig. 4D). However, when charging effects are sufficiently large, the system instead traverses across a single energy landscape that includes both the dense cluster state and the partial film (Fig. 4E) to produce a smooth, sigmoidal variation in the surface coverage with chemical potential. The effect of charging on the energy landscape is similar to that obtained by simple, supercritical simulations (i.e., no charging effects, ε < ε_crit_), but the corresponding microstructures show more complexity.

**Fig. 5.**
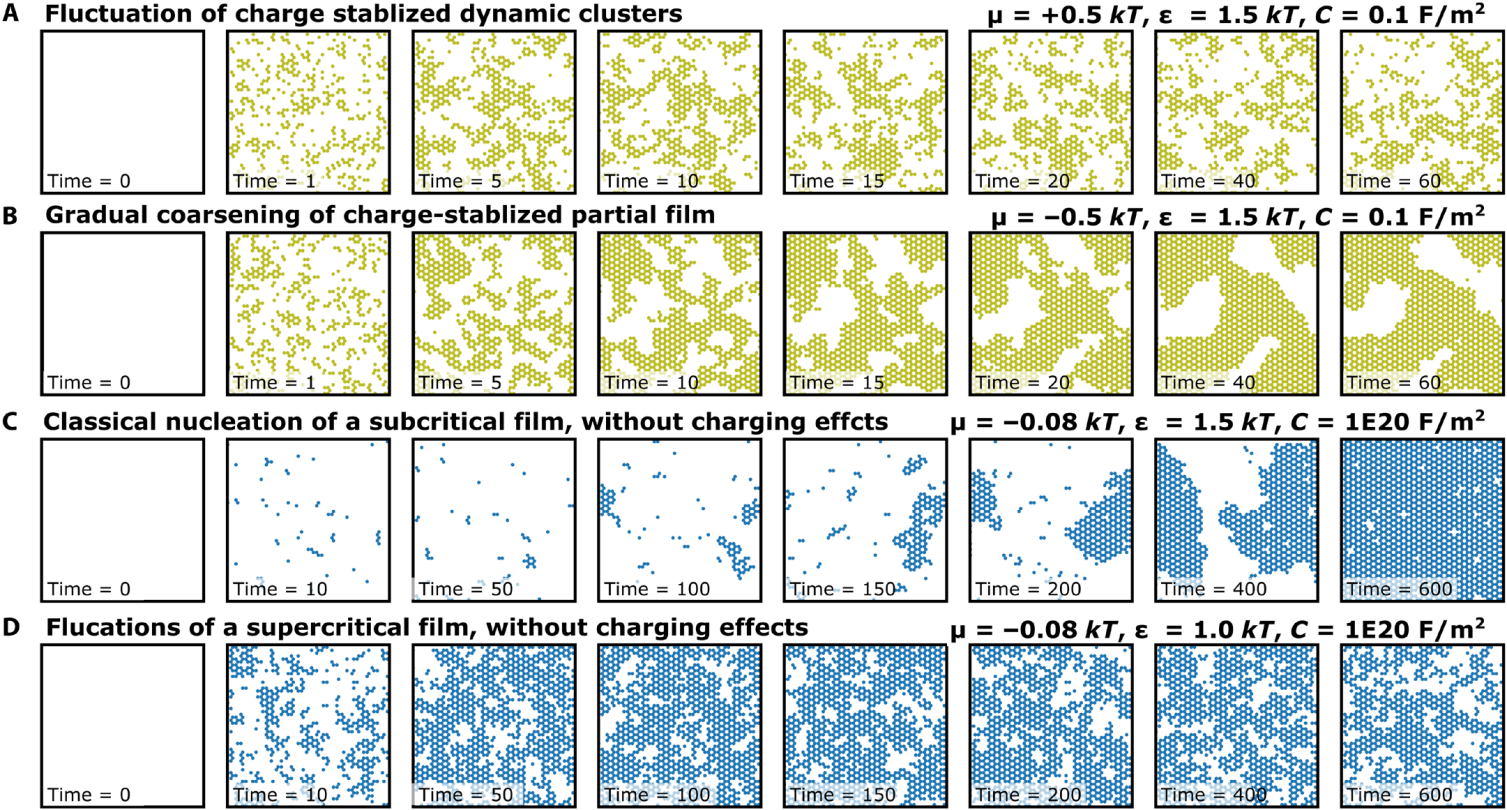
Impact of charging on KMC-predicted surface dynamics. (**A**) Time evolution of a system with *C* = 0.1 F/m^2^, ε = 1.5 *kT*, and μ = +0.5 *kT*, showing rapid formation of dynamically fluctuating clusters. (**B**) Time evolution of a system with *C* = 0.1 F/m^2^, ε = 1.5 *kT*, and μ = −0.5 *kT*. Rapid formation of clusters is followed by gradual growth and coalescence into a continuous partial film. (**C**) Time evolution of a subcritical system with *C* = 1 × 10^20^ F/m^2^ (no significant charging), ε = 1.5 *kT*, and μ = −0.08 *kT*. After nucleation from a sparsely covered surface, a single island grows to cover the entire surface. (**D**) Time evolution of a supercritical system with *C* = 1 × 10^20^ F/m^2^, ε = 1.0 *kT*, and μ = −0.08 *kT*. The surface is quickly covered by a film that continues to fluctuate rapidly and does not coarsen.

The simulations also reproduce the basic kinetic behavior observed with AFM (Fig. 5). In the μ > 0 regime (Fig. 5A), the simulations predict that clusters should fluctuate rapidly in size and shape, consistent with experiments from *T* = 40° to 50°C. In contrast, in the μ < 0 regime (Fig. 5B), the simulations predict an initially rapid increase in coverage (as electrostatic forces and chemical potential forces work in tandem to rapidly drive high quantities of aluminum to the surface), followed by a gradually slowing coarsening process after overcharging is achieved, similar to the coarsening process observed in AFM at *T* = 65°C. This good agreement between simulations and experiment could not be achieved with simpler models that neglect charging. The subcritical charging-free models produced classical rare-event nucleation processes (Fig. 5C) in which high intermediate coverages could not persist. In addition, while the supercritical charging-free models could produce high intermediate coverages (Figs. 3E and 5D), they could not explain the transition from rapid fluctuations at low *T* (high μ) to gradual coarsening at high *T* (low μ). Instead, the simple supercritical models incorrectly predicted that high-coverage films would fluctuate rapidly and indefinitely without coarsening or slowing. Surface charging seems uniquely capable of explaining the full range of behavior because of its ability to have different effects as the system transitions from undercharged to overcharged.

## DISCUSSION

The above findings reveal a diversity of transient structures as gibbsite films grow on the mica surface. These results confirm certain essential aspects of CNT, such as the existence of fluctuating clusters whose populations decay exponentially with size. However, we ultimately find that gibbsite films form via a complex, nonclassical pathway marked by a marked deviation of the clusters’ energy landscape from the expectations of CNT.

These deviations can be explained by accounting for the impact of surface electrostatic forces. These forces simultaneously modulate the concentration of ions at the surface and change their tendency to associate into clusters. When the surface is undercharged before film formation, electric forces drive more aluminum to the surface but favor its dissociation into smaller clusters. Consequently, formation of gibbsite at the mica interface involves assembly from a dense layer of aluminum clusters, rather than the classical process of forming rare clusters from a dilute solution, as occurs in bulk solution. This surface-driven phenomenon allows crystallization to proceed via a gradual evolution of states resembling those seen in supercritical or near-critical systems, and the process of forming critically sized clusters to overcome classical nucleation barriers no longer plays a role. This is reflected by a change in the system’s free energy landscape from a system with two distinct phases to a system that undergoes microphase separation to create a single nanostructured surface phase.

Electrostatic forces also explain why the resulting films retain a network of persistent, nanometer-scale gaps, because this structure is stabilized by the competition between edge energies (which favor coarsening) and longer-range electrostatic forces (which favor local charge neutrality). The characteristic length scale associated with this patterning depends on the specific features of the gibbsite-mica system. This length scale is not directly accessible through our model because the electrostatic forces have been approximated using mean-field methods. We anticipate that the length scale will be related to the Debye length, which is estimated to be ~4 nm for this system. Although more work will be needed to refine the long-range interaction terms, in the future, we hope to move beyond a mean-field treatment of the electrostatic forces to consider models that treat the long-range electrostatic forces explicitly. However, we note that many theoretical studies by other groups have shown that 2D frustrated systems (specifically those where short-range attractive bonding competes with long-range repulsive interactions) can reproduce the types of structures and phenomenology we observe here (*39*–*43*).

The broader implication of our simulations is that the clusters, islands, and gaps are linked through electrostatic forces, such that their stability depends intimately on the properties of the clusters that surround them. Thus, the gibbsite clusters and gaps are not independent phases but microdomains of a single nanostructured phase. This phase is analogous to the well-known striped, cylindrical, or gyroid phases that are widely observed in block copolymer and surfactant systems, which are also single-phase systems composed of distinct microdomains (*44*, *45*). Microphase separation of block copolymers occurs because the demixing of hydrophobic and hydrophilic blocks is prevented by covalent linkages between the blocks. Notably, past work by Wu *et al*. (*43*) has demonstrated that microphase separation of surfactants and polymers can be modeled through an electrostatic analogy, in which electrostatic forces play the role of covalent linkages. For our gibbsite films, the separation into independent phases appears to be inhibited by actual electrostatic forces, and our observation of microphase separation, thus, provides a physical realization of that analogy.

The emergence of this nanostructured phase has especially important consequences for the precipitation pathway because it is associated with the disappearance of the miscibility gap. Microphase-separated phases can display properties that are intermediate between fully disordered (such as random adsorption of ions) and fully ordered (such as the formation of a continuous gibbsite film). Our simulations indicate that diverse structures ranging from sparse clusters to continuous partial films can all exist within a single region of the phase boundary, a result that is consistent with similar simulations by Almarza *et al*. (*40*). Consequently, the film can evolve continuously from a low-coverage state of isolated fluctuating clusters to a high-coverage nanostructured network, while always remaining within a single region of the phase diagram. The process of precipitation is then limited by the kinetics of coalescence and coarsening rather than the formation of critically sized clusters, and there is no need to overcome a classical nucleation barrier.

This work highlights the complex and marked influences that interfaces and electric fields can have on crystal nucleation and growth and provides guidance on how one might promote or inhibit crystallization by tailoring surface charge states. These insights were facilitated by direct observation of the crystallization process, demonstrating that the era of high-speed, atomically resolved in situ AFM holds great promise for investigating processes of mineral phase transformation.

## MATERIALS AND METHODS

### AFM imaging

Clusters were imaged with a Cypher ES AFM (Oxford Instruments) with perfusion cell, heating-cooling stage, and ultrasharp (2-nm nominal radius) high-density carbon probes (USC-F1.5-k0.6, NanoWorld). Probes were rinsed with isopropanol and mounted with a PETE clamp and screw. A 10-mm mica sample disc (grade V1 muscovite, Ted Pella) was mounted on a 10-mm gold-plated sample puck (Ted Pella) with crystal bond adhesive. The sample was rinsed with water and tape-cleaved immediately before imaging to expose a fresh surface and then mounted in a small envelope of Kapton to prevent solvent evaporation (see fig. S1).

Room-temperature images in a 100-μl droplet of 10 mM HCl were obtained to optimize conditions; then, the cell was flushed with 2 ml of 0.2 mM HCl + 1 mM AlCl_3_ solution, delivered via syringe pump through polytetrafluoroethylene tubing, and the Kapton envelope was flooded with 1 ml of 0.2 mM HCl + 1 mM AlCl_3_ solution for final imaging.

The temperature was increased to image at 30°, 40°, 42.5°, 45°, and 50°C, and imaging was terminated at 55°C. We used amplitude modulation feedback, with a drive frequency of 1.07 MHz, a free amplitude of 820 pm, and a set point of 760 pm (with minor adjustments to maintain image quality). Image sizes of 60 nm by 120 nm (256 pixels by 512 pixels) and line rates of 13 Hz produced images at 20-s intervals.

AFM images of film growth (65°C) were obtained using a similar procedure, but using a USC probe operated at 1.41 MHz, a scan size of 120 nm by 120 nm (512 pixels by 512 pixels), and line rates of 20 Hz, producing images at 26-s intervals.

### Solution preparation

Solutions were prepared from TraceSELECT hydrochloric acid (Fluka), 99.999% pure aluminum chloride hydrate (Sigma-Aldrich), and OmniTrace Ultra water (EMD Millipore), as described in (*15*).

### Image processing and analysis

AFM height-channel images were coaligned to subpixel accuracy with SimpleElastix (*46*) using affine transforms to correct for drift distortion. Images were then line-flattened, contrast-normalized with respect to their SD, and destreaked using an anisotropic 3D median filter (kernel size of 7 by 5 by 3, vertical × horizontal × time).

The long-wavelength background (estimated from a 3D Gaussian-blurred image with a kernel size of 12 by 12 by 1) was subtracted, and images were segmented using a height threshold of 0.8 SDs. Clusters were assigned by identifying spatially connected regions in the segmented image. Example for images for thresholding is shown in fig. S2, and height histograms used to establish thresholding criteria are shown in fig. S3.

### Trajectory analysis

Cluster size trajectories were produced by linking clusters whose spatial footprint overlapped in consecutive frames. Splitting or merging events were recorded wherever clusters were linked to multiple clusters in an adjacent frame, with continuous trajectories linking the clusters with the greatest overlap.

Monodisperse ensembles were prepared by identifying clusters that fell within a defined size window at any time during the experiment. Subsequent trajectories, starting from the first moment a cluster fell into the window, were averaged to generate an ensemble trajectory. Clusters that went to zero size were retained as a zero-size cluster for purposes of calculating ensemble averages. Trajectory data are summarized in fig. S4.

We extracted microscopic physical parameters by measuring the initial rate of change in ensemble average size, $d\bar{n}/\mathrm{dt}$, and the rate of change in ensemble size variance, *d*σ^2^/*dt*, in terms of monomer units, for the ensembles of interest. We then estimate the average rate of monomer addition/subtraction as

$$k_{\text{avg}}=\frac{1}{2}\frac{d\sigma^{2}\;}{\mathrm{dt}}$$ (3)

and the slope of the energy landscape as

$$\frac{d∆G_{n}}{\mathrm{dn}}=(\frac{d\sigma^{2}\;}{\mathrm{dt}}+\frac{d\bar{n}\;}{\mathrm{dt}})/(\frac{d\sigma^{2}\;}{\mathrm{dt}}-\frac{d\bar{n}\;}{\mathrm{dt}})$$ (4)

The derivation of these expressions is demonstrated in section S4, they are validated against simulations in section S5, and their application to experimental data is shown in section S6.

### Monte Carlo simulation

Monte Carlo simulations used a standard kinetic Metropolis algorithm, implemented in Python. We simulated a 4096-site (*n*_sites_ = 4096) honeycomb lattice with periodic boundary conditions that represents ~480 nm^2^ of mica surface. Because the electrostatic forces are treated in a mean-field manner, the simulation size is an important physical parameter and is chosen to correspond to the expected length scales of electrostatic interactions. At each iteration, a site was randomly selected for potential change in occupancy to approximate to the random addition/subtraction of aluminum ions on the mica surface. The simulation state was saved every 4096 attempts. The charging-free simulations (shown in Fig. 3) were run for 4,096,000 iterations (1000 states), and simulations with charging (shown in Figs. 4 and 5) were run for 1,638,400 iterations (400 states) to provide burn-in, and equilibrium coverages were determined from the final 100 states.

The system energy is calculated as

$$E=E_{\text{occ}}+E_{\text{edge}}+E_{\text{charge}}$$ (5)

The site occupancy energy is *E*_occ_ = *n*_occ_ ∙ μ/*kT*, where *n*_occ_ is the number of occupied sites. Each occupied site has three edges, which are unbonded if the corresponding neighboring site is unoccupied. The system edge energy is *n*_edge_ ∙ ε/*kT*, where *n*_edge_ is the number of unbonded edges. The surface charging energy is calculated as *E*_charge_ = (*n*_sites_ ∙ *q*_site_ + *n*_occ_ ∙ *q*_occ_ + *n*_edge_ ∙ *q*_edge_)^2^/2*C*_sys_*kT*.

Here, *n*_sites_ is the total number of sites in the system (including both occupied and unoccupied). We used values of *q*_site_ = −0.25 *e* (reflecting the negative charge of the mica surface) and *q*_occ_ = + 0.333 *e* and *q*_edge_ = + 0.333 *e* (reflecting the positive charge of aluminum ions). A key aspect of the chosen parameters is that *q*_occ_ has a larger magnitude than *q*_site_, such that overcharging will occur and prevent the formation of continuous films. It is also important that *q*_edge_ be positive, so that effective edge tension will change dynamically in response to surface charge. *C*_sys_ is the system capacitance, where values of 300, 3000, 30,000, and 3 × 10^23^ e/V were used to reflect surface capacitance densities of 0.1, 1, 10, and 1 × 10^20^ F/m^2^, respectively. The first three values reflect the range of commonly encountered surface capacitances in constant capacitance and triple-layer models (*35*, *38*), while the final value was used to approximate the limiting case of zero charging effects. *kT* was approximated as 0.0257 eV in all simulations.

## Supplementary Materials

### This PDF file includes:

Sections S1 to S11 Figures S1 to S8 References
