# Supplementary material for: Hydroxide films on mica form charge-stabilized microphases that circumvent nucleation barriers
Source: Sci Adv. 2022 Sep 2;8(35):eabn7087. doi: 10.1126/sciadv.abn7087 (PMC10848954; doi:10.1126/sciadv.abn7087)
Supplement: Supplementary file 1 — Sections S1 to S11 Figures S1 to S8 References [file sciadv.abn7087_sm.pdf]

Science Advances
AAAS

Supplementary Materials for

Hydroxide films on mica form charge-stabilized microphases that circumvent nucleation barriers

Benjamin A. Legg *et al.*

Corresponding author: James J. De Yoreo, james.deyoreo@pnnl.gov; Benjamin A. Legg, benjamin.legg@pnnl.gov

Sci. Adv. **8**, eabn7087 (2022)
DOI: 10.1126/sciadv.abn7087

The PDF file includes:

Sections S1 to S11
Figures S1 to S8
Legends for movies S1 to S5
Legends for data S1 and S2
References

Other Supplementary Material for this manuscript includes the following:

Movies S1 to S5
Data S1 and S2

1. Schematic of AFM Cell

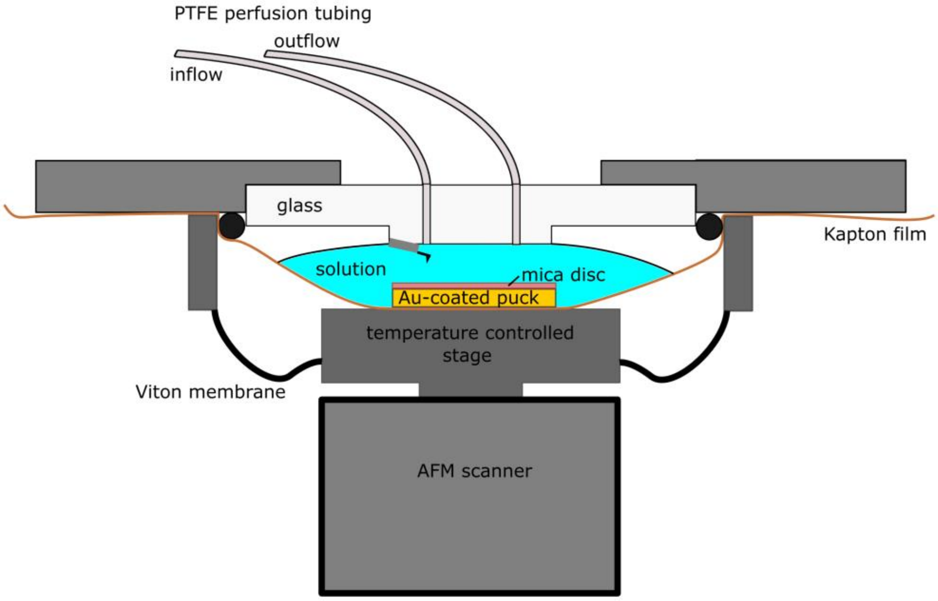

Fig. S1. Schematic of AFM cell

Schematic of in situ cell used for AFM imaging in Cypher VRS (not to scale). The perfusion enabled tip-holder and temperature-controlled sample stage are commercially available accessories. A thin Kapton film (shown in orange) was added for these experiments to enable safe handling of mL-quantities of reactive solution at elevated temperature. The configuration of the cell with the Kapton film reduces the head-space available for evaporation and causes any water that evaporates to condense and return to the solution. The Viton membrane provides secondary containment to protect sensitive parts of the instrument, in case the Kapton film leaks. Rather than a traditional steel sample puck, a gold-coated puck was used to minimize the potential for corrosion and contamination of the solution. The AFM-probe was also mounted using a PEEK tip clamp, instead of a standard steel tip clamp, to further reduce the potential for corrosion.

2. AFM Image Processing Example

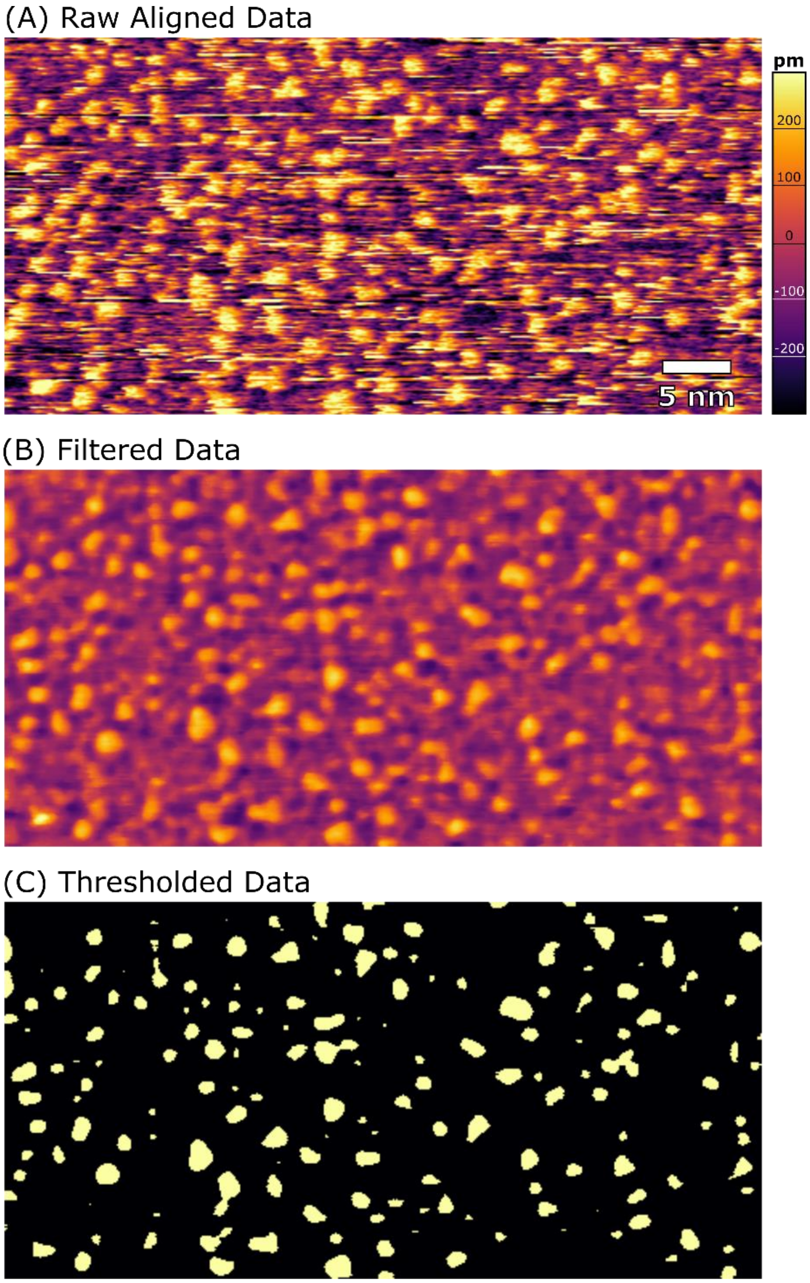

Fig. S2. AFM image processing example.
Sample frames demonstrating the image processing stages for fluctuating cluster movies. (A) Raw AFM height-channel image after frame-to-frame alignment (cropped image). (B) Data after filtering to remove streaking, image noise, and long-wavelength background, as described in methods (C) Final thresholded data, used to calculate cluster size distributions and size trajectories.

3. AFM Height Thresholding

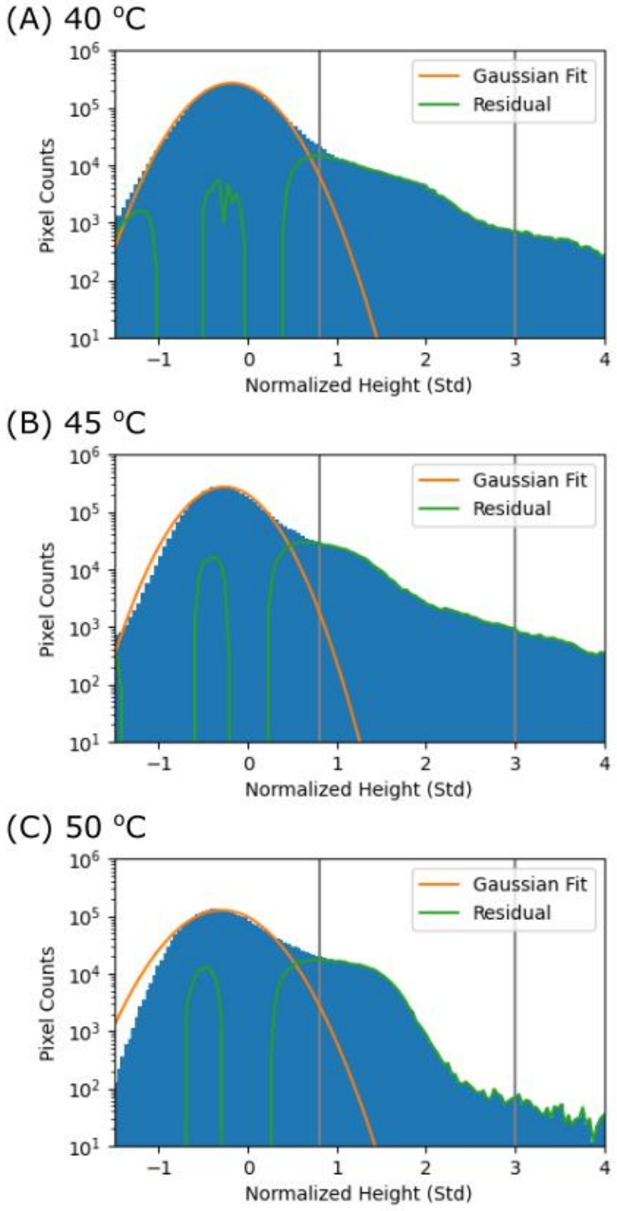

Fig. S3. AFM height thresholding.
Pixel height histograms of the processed movies are shown for each condition (A) 40 °C, (B) 45 °C, and (C) 50 °C. The pixel heights are normalized to units of sample standard deviation. The orange curve is a Gaussian fit to the full histogram. Its width primarily reflects the substrate height variation, which is influenced by hydration structure and imaging noise. The green curve shows the fitting residual. A distinct shoulder centered between 1 and 2 standard deviations reflects the presence of surface adsorbates. Vertical lines at 0.8 and 3 standard deviations mark the range of pixel values that are classified as Al-surface clusters in our analysis. Regions below 0.8 standard deviations are classified as bare surface, and those above 3 standard deviations are classified as 3D particles.

4. Cluster Size Trajectory Compilation

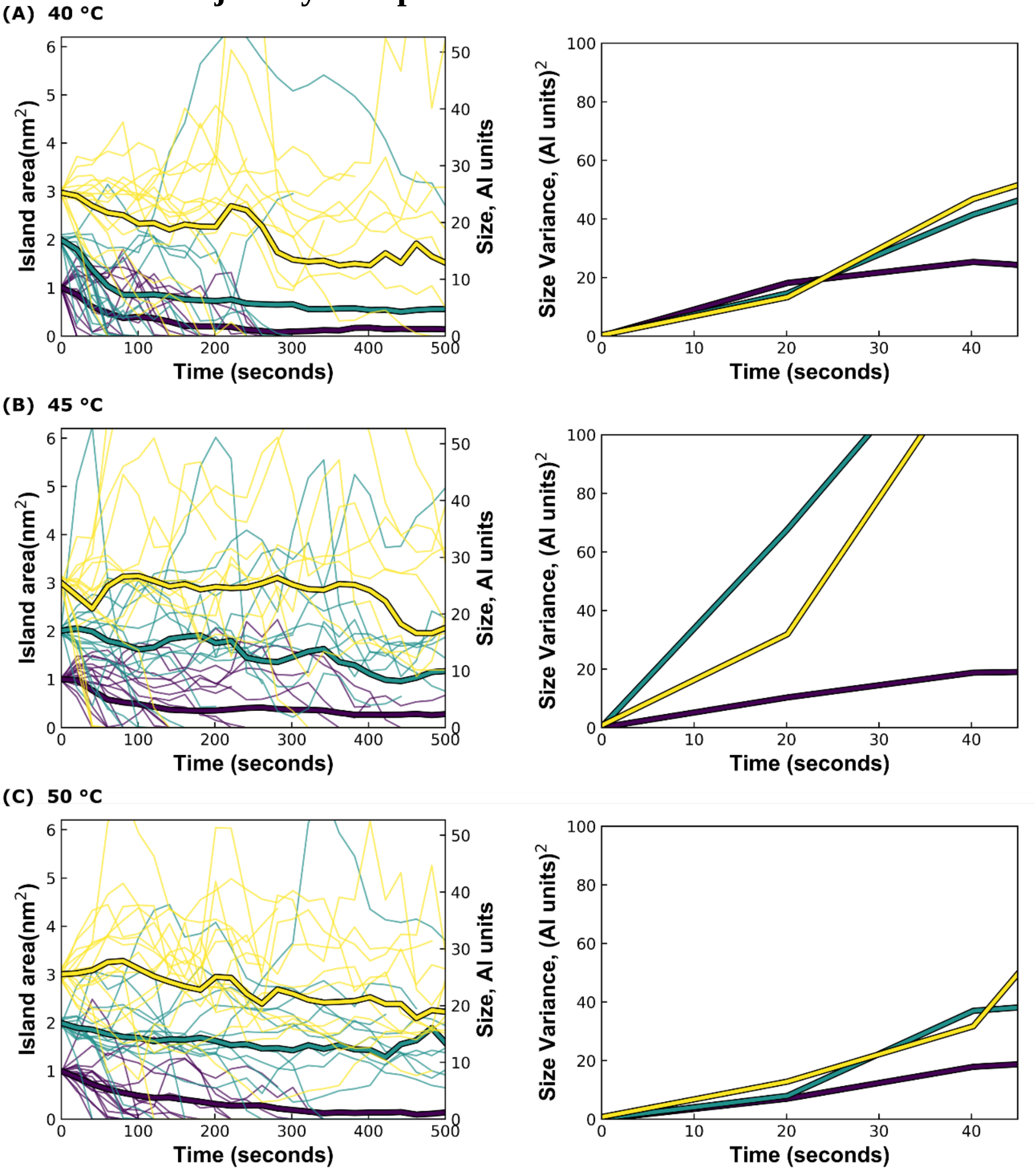

Fig. S4. Experimental cluster size trajectories.

Plots in left-column show trajectories of ensemble-average size for initially monodisperse ensembles of clusters (heavy lines, outlined in black), along with size trajectories for selected individual clusters from those ensembles, at temperatures of (A) 40 °C (B) 45 °C, and (C) 50 °C. Plots in right-column show development of ensemble variance. The variance initially increases with time, but it can decrease after long time periods due the reduction of the average cluster size and the disappearance of small clusters. Ensembles were prepared with initial sizes near 3.0 nm^2 (yellow), 2.0 nm^2 (cyan), or 1.0 nm^2 (purple).

5. Size Fluctuation Analysis: Theoretical Development

We assume that cluster fluctuations follow a biased random walk through size-space, facilitated by the addition and subtraction of monomer units (where each unit is 0.1177 nm^2). The evolution of cluster size distributions is thus assumed to depend upon the size-dependent rate constant for monomer addition, k_{on}, and monomer subtraction, k_{off}.

The evolution of average cluster size (in monomer-units) will reflect the difference between these rate constants:

Eq. S1 $$\frac{d\bar{n}}{dt} = k_{on} - k_{off} = \Delta k$$

And if the addition and subtraction events follow Poisson statistics, the size variance, σ^2, of an initially monodisperse ensemble will increase at a rate that depends on the sum of these rate constants:

Eq. S2 $$\frac{d\sigma^2}{dt} = k_{on} + k_{off} = 2k_{avg}$$

Thus, the average rate of monomer addition and subtraction may be obtained experimentally by measuring the rate of change in ensemble variance, as follows:

Eq S3: $$k_{avg} = \frac{(k_{on}+k_{off})}{2} = \frac{1}{2}\frac{d\sigma^2}{dt}$$

Furthermore, elementary statistical mechanics principles allow us to relate the slope of the cluster energy landscape to the ratio of k_{on} and k_{off} as follows:

Eq. S4: $$\frac{d\Delta G_n}{dn} \approx -kT \ln\left(\frac{k_{on}}{k_{off}}\right)$$

Substitution of variables allows us to express this value in terms of experimental observables.

Eq. S5: $$\frac{d\Delta G_n}{dn} = -kT \ln\left[\left(\frac{d\sigma^2}{dt} + \frac{d\bar{n}}{dt}\right) / \left(\frac{d\sigma^2}{dt} - \frac{d\bar{n}}{dt}\right)\right]$$

Importantly, this expression holds even for non-equilibrium distributions and clusters above the critical size. (In contrast, Eq. 1 of the main text provides a direct assessment of ΔG_n, but is only accurate for distributions that are near-equilibrium).

6. Size Fluctuation Analysis: Benchmarking against KMC Models

In order to validate the size-fluctuation analysis in the previous section, the analytical expressions were tested against synthetic ensemble trajectories, generated using a standard rejection-free kinetic Monte Carlo (KMC) algorithm, implemented in Python. These simulations assume that the particles follow a biased random walk across a classical cluster energy landscape, where the probability of a growth event occurring at any step is $k_{on}/(k_{on}+k_{off})$ and the probability of a dissolution event is $k_{off}/(k_{on}+k_{off})$. The time-lapse between each step is determined as $\ln(1/u)\,/(k_{on}+k_{off})$, where u is a randomly generated number between 0 and 1.

The assumed energy landscapes are expressed as follows:

Eq. S6: $$\Delta G_n = \tau \cdot 2\sqrt{\pi n} + n\,\Delta\mu$$

For a cluster of size n, the monomer on and off rates are assumed to conform to the following expressions:

Eq. S7a: $$k_{on} = k_0\sqrt{n}$$

Eq. S7b: $$k_{off} = k_0\sqrt{n}\ \exp((\Delta G_n - \Delta G_{n-1})/kT)$$

Several simulated ensemble size-trajectories and size-variance trajectories are shown in Fig. S5, for two classical energy landscapes. First we simulate a 'gentle' landscape with values of $\tau = 0.9$ and $\Delta\mu$ = -0.225 (corresponding to a critical energy barrier of 11.3 kT at n = 50, and maximum slope of $d\Delta G_n/dn =$ 2.96 kT), and second we simulate a 'steep' landscape with values of $\tau = 9.0$ and $\Delta\mu$ = -2.25 (critical energy barrier of 113 kT at n = 50 and maximum slope of $\Delta G_n/dn = 29.6\ kT$). (Note that the symbols k_{on}, k_0 and k_{off} refer to rate constants, while the symbol without a subscript, k, is the Boltzmann constant).

These simulations produce ensemble dynamics that share several characteristics with AFM experimental results. Individual trajectories can show significant variation, and the size variance of the ensemble always initially increases (in accordance with Eq. S2), but whether the ensemble average size increases depends upon whether the ensemble is poised above or below the critical size, in accordance with Eq. S1.

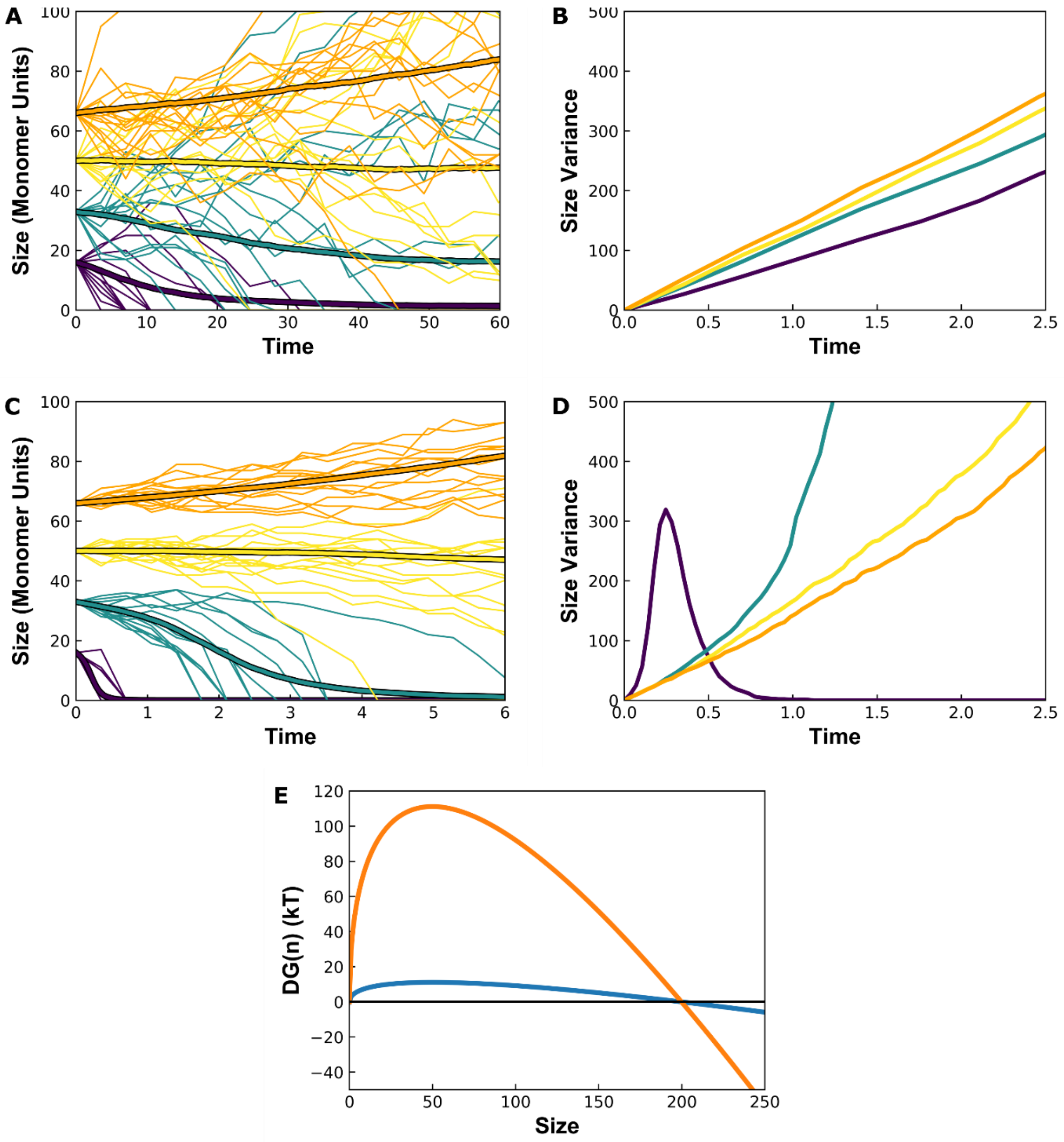

Fig. S5. Simulated cluster size trajectories.

(A) KMC simulations of cluster trajectories when the critical energy barrier is 11.1 kT. Initial sizes are 16 atoms (purple), 33 (cyan), 50 (yellow), and 66 (orange). (B) Corresponding increase in variance of cluster ensembles with time. Slower rate of increase for the purple curve (initial size of 16) is because reaction rates scale with cluster edge-length. (C) KMC simulations of cluster trajectories when critical size is 111 kT, (D) Corresponding increase in variance of cluster ensembles with time. (E) Plots of the corresponding classical energy landscapes, with the gentle landscape in blue, and the steep landscape in orange.

Closer examination of the data in Fig. S5 allows us to compare the initial values of $d\sigma^2/dt$ and $d\bar{n}/dt$ generated in simulations with the corresponding values predicted by Eq. S1 and Eq. S2. As shown in Fig. S6, we generally find good agreement, although there are some noticeable, systematic deviations for very small clusters. These can be attributed to curvature in the energy landscape (which can either stretch or focus the cluster size distributions, depending on the direction of curvature), and boundary effects that emerge when significant numbers of clusters reach zero-size. Thus, we conclude that Eq. S3 and Eq. S5 can be used to estimate k_{avg}, and $d\Delta G_n/dn$ from ensemble data, so long as the cluster ensembles are sufficiently large, and the elapsed time between measurements is short enough that boundary-effects can be neglected.

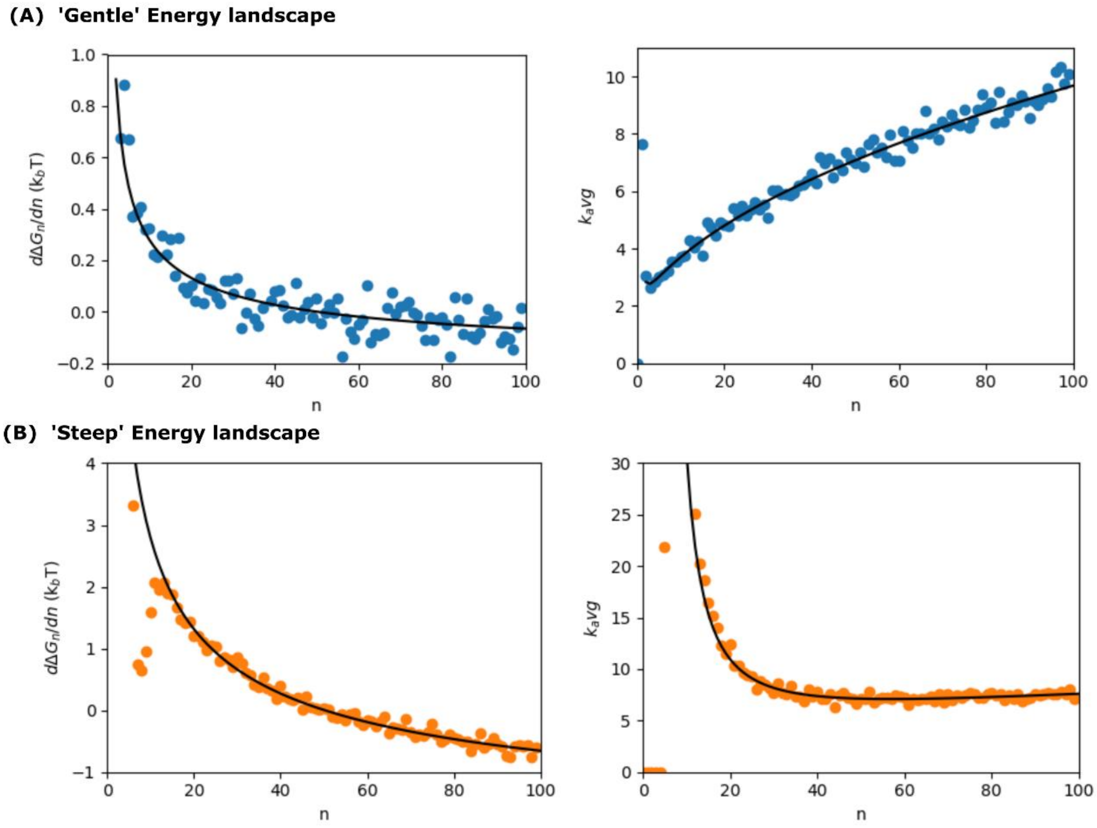

Fig. S6. Benchmarking methods for estimating properties of the cluster energy landscape and rates of atomic attachment/detachment with KMC simulations.
Plots on left compare the known size-dependent slope of the energy landscape (black line) that is used as input to KMC simulations with the values that are recovered by analyzing the resulting KMC trajectory ensembles with Eq S5 (are shown in colored symbols). Plots on right compare the known input values of k_{avg} (black line), with values recovered by application of Eq S3.

7. Size Fluctuation Analysis: Application to Experiment

Fig. S7 shows a compilation of average rates for change in ensemble variance (A) and size (B) and applies Eq. S4 and Eq S5 to estimate the k_{avg} (C) and $d\Delta G_n/dn$ (D). In order to reflect the physics of cluster growth/dissolution, we plot k_{avg} normalized with respect to $3\sqrt{n}$, which reflects the approximate number of reactive sites around the perimeter of a cluster. The results indicate that k_{avg} is on the order of 0.01 s^{-1} to 0.1 s^{-1} per reactive site. This is consistent with previous observations that hydrolyzed aluminum ions have lifetimes of tens to hundreds of seconds on the mica surface.[19] Interestingly, the rate shows little dependence on temperature. Furthermore, the slopes of $d\Delta G_n/dn$ are estimated to be on the order of 0-0.1 kT. This reflects a gentle energy landscape with low driving forces for dissolution (even slightly gentler than predicted from population counting).

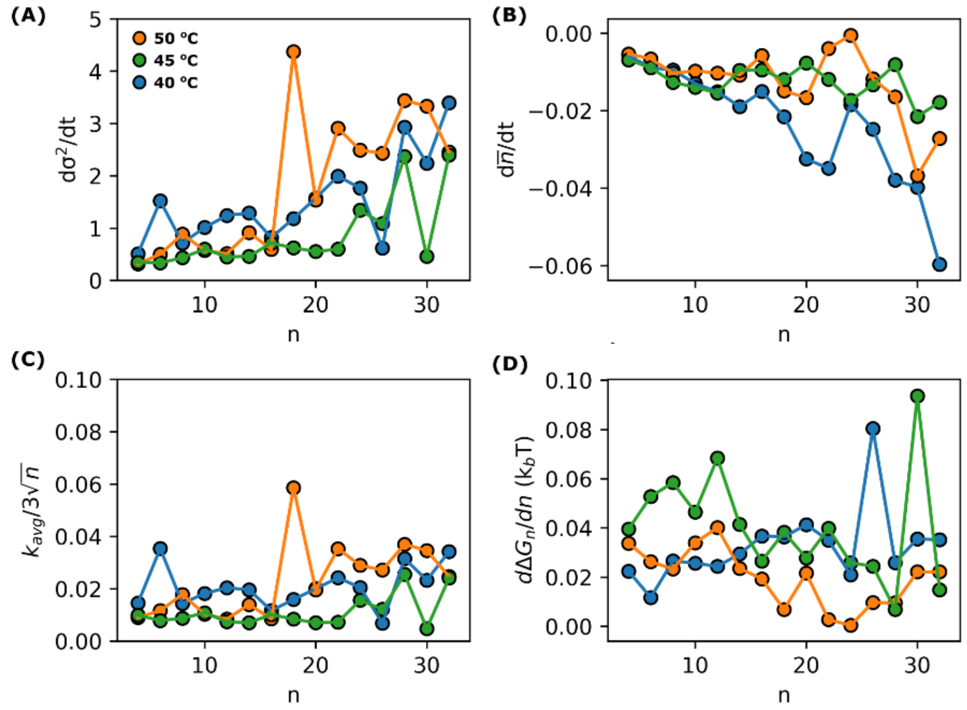

Fig. S7. Estimating cluster energy landscape parameters and rates of atomic attachment/detachment from experimental cluster dynamics.
(A) Plot of the rate of increase for cluster ensemble variance, as a function of ensemble size, as determined by experiments at 40, 45, and 50 °C. (B) Corresponding plots of the rate of change for cluster-ensemble average size. (C) Estimated average attachment/detachment rate per edge-site, as a function of clusters size, as calculated from the cluster ensemble dynamics using Eq. S3. (D)

Calculated slope of the energy landscape, as a function of cluster size, calculated from the cluster ensemble dynamics using Eq. S5.

8. Estimated Edge Tensions of Bulk Gibbsite

Edge tensions of bulk gibbsite in aqueous solution can be estimated in several ways. DFT-based calculations suggest surface energies ~700 mJ/m^2 for the (110) and ~1000 mJ/m^2 for the (100) faces. When normalized for the layer thickness of ~ 4.9 Å, this predicts edge energies between 350 and 500 nJ/m. However, such values are likely to significantly overestimate nucleation barriers, and effective surface/edge tensions can be drastically reduced in aqueous solution due to coadsorption of ions.[47] Expressions Ref. 47 allow us to estimate the surface tension of gibbsite at roughly 60 mJ/m^2, based on its bulk solubility (which is roughly 2.5 mM in the pH 6, chloride-containing solutions considered here). Neglecting face-dependent variation, this would lead to much lower edge tensions on the order 30 nJ/m. In fact, this neglection of face-dependance is likely conservative, and will probably produce a low estimate, since the edges are typically assumed to be higher in energy than the basal plane. However, even this estimate is still orders of magnitude above the observed values, for which our fits assumed a total edge energy proportional to 3.6 $kT\sqrt{n}$ (where n is the number of aluminum ions in the cluster). Then, given that circumference of a cluster can be approximated as ~ 1.22 $\sqrt{n}$ nm, and given that kT is on the order of $4 \cdot 10^{-21}$ J (although increasing slightly with temperature), this corresponds to an extraordinarily low experimentally observed edge tension of on the order of just 0.012 nJ/m, several orders of magnitude smaller than expected based on bulk surface tensions.

9. Structure of Gibbsite Film

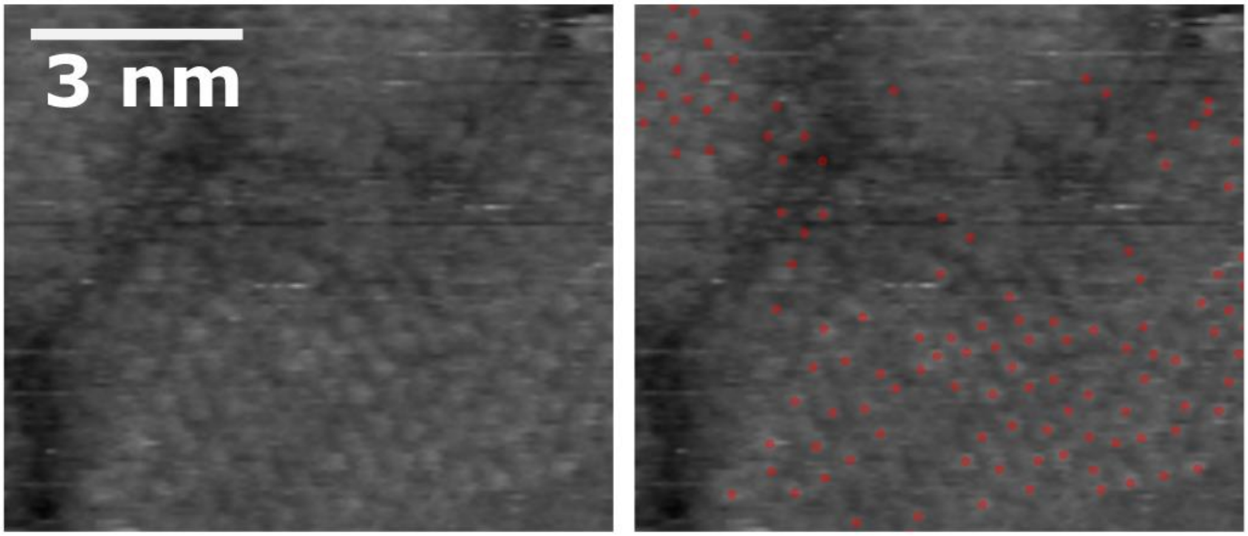

Fig. S8. High resolution images of a gibbsite film.
High resolution image of an aged gibbsite film. The left is the original AFM image, with sub-nanometer structure clearly visible. The right is a modified image, with red-dots indicating the location of high-features in the image. Although distinct rows of atoms are visible, the annotated imaging shows clearly that the spacing between atoms is imperfect and irregular. This points to poor crystallinity, that will be associated with the presence of broken internal bonds that are capable of carrying additional surface charge.

10. Description of Supplementary Movies

Movie S1. Fluctuating clusters at 40 °C

Time-resolved AFM movie of fluctuating sub-critical clusters at the muscovite-water interface. Obtained at 40 °C, using a USC-F1.2-k7.3 probe, in an aqueous solution of 1 mM $AlCl_3$ and 0.2 mM HCl. Upper-half is the filtered AFM movie. Lower half is the thresholded data, used to determine cluster-size distributions and trajectories. Time resolution is 10 seconds per frame.

Movie S2. Fluctuating clusters at 45 °C

Time-resolved AFM movie of fluctuating sub-critical clusters at the muscovite-water interface. Obtained at 45 °C, using a USC-F1.2-k7.3 probe, in an aqueous solution of 1 mM $AlCl_3$ and 0.2 mM HCl. Upper-half is the filtered AFM movie. Lower half is the thresholded data, used to determine cluster-size distributions and trajectories. Time resolution is 10 seconds per frame.

Movie S3. Fluctuating clusters at 50 °C

Time-resolved AFM movie of fluctuating sub-critical clusters at the muscovite-water interface. Obtained at 50 °C, using a USC-F1.2-k7.3 probe, in an aqueous solution of 1 mM $AlCl_3$ and 0.2 mM HCl. Upper-half is the filtered AFM movie. Lower half is the thresholded data, used to determine cluster-size distributions and trajectories. Time resolution is 10 seconds per frame.

Movie S4. Fluctuating clusters at 55 °C

Time-resolved AFM movie of fluctuating sub-critical clusters at the muscovite-water interface. Obtained at 55 °C, using a USC-F1.2-k7.3 probe, in an aqueous solution of 1 mM $AlCl_3$ and 0.2 mM HCl. Upper-half is the filtered AFM movie. Lower half is the thresholded data, used to determine cluster-size distributions and trajectories. Time resolution is 10 seconds per frame. (Note change of scale relative to Movies S1-3)

Movie S5. Film growth at 65 °C

Time-resolved AFM movie of island coalescence to produce an extended aluminum hydroxide film at the muscovite-water interface. Obtained at 65 °C, using a USC-F1.2-k7.3 probe in an aqueous solution of 1 mM $AlCl_3$ and 0.2 mM HCl.

11. Description of Supplementary Code

Code S1. Nucleus Fluctuation Kinetic Monte Carlo Simulations.

Zip file containing a readme.txt, documented Python source code for generating kinetic Monte Carlo trajectories of nucleus fluctuations on a classical energy landscape, and sample output data.

Code S2. Monte Carlo Honeycomb Lattice Gas Simulations.

Zip file containing a readme.txt file, documented Python source code for conducting Monte Carlo lattice gas simulations on a 2D honeycomb lattice, and sample output data.

REFERENCES AND NOTES

1. J. W. Gibbs, On the equilibrium of heterogeneous substances. *Am. J. Sci.* **16**, 441 (1878).

2. J. J. De Yoreo, P. G. Vekilov, Principles of crystal nucleation and growth. *Rev. Mineral. Geochem.* **54**, 57–93 (2003).

3. K. F. Kelton, A. L. Greer, *Nucleation in Condensed Matter* (Elsevier, 2010)

4. R. Becker, W. Döring, The kinetic treatment of nuclear formation in supersaturated vapors. *Ann. Phys.* **24**, 719–752 (1935).

5. P. G. Vekilov, Dense liquid precursor for the nucleation of ordered solid phases from solution. *Cryst. Growth Des.* **4**, 671–685 (2004).

6. D. Gebauer, A. Völkel, H. Cölfen, Stable prenucleation calcium carbonate clusters. *Science* **322**, 1819–1822 (2008).

7. A. F. Wallace, L. O. Hedges, A. Fernandez-Martinez, P. Raiteri, J. D. Gale, G. A. Waychunas, S. Whitelam, J. F. Banfield, J. J. De Yoreo, Microscopic evidence for liquid-liquid separation in supersaturated $CaCO_3$ solutions. *Science* **341**, 885–889 (2013).

8. W. J. E. M. Habraken, J. Tao, L. J. Brylka, H. Friedrich, L. Bertinetti, A. S. Schenk, A. Verch, V. Dmitrovic, P. H. H. Bomans, P. M. Frederik, J. Laven, P. van der Schoot, B. Aichmayer, G. de With, J. J. De Yoreo, N. A. J. M. Sommerdijk, Ion-association complexes unite classical and non-classical theories for the biomimetic nucleation of calcium phosphate. *Nat. Commun.* **4**, 1507 (2013)

9. J. J. De Yoreo, P. U. P. A. Gilbert, N. A. J. M. Sommerdijk, R. L. Penn, S. Whitelam, D. Joester, H. Zhang, J. D. Rimer, A. Navrotsky, J. F. Banfield, A. F. Wallace, F. M. Michel, F. C. Meldrum, H. Cölfen, P. M. Dove, Crystallization by particle attachment in synthetic, biogenic, and geologic environments. *Science* **349**, aaa6760 (2015).

10. K. Cao, J. Biskupek, C. T. Stoppiello, R. L. McSweeney, T. W. Chamberlain, Z. Liu, K. Suenaga, S. T. Skowron, E. Besley, A. N. Khlobystov, U. Kaiser, Atomic mechanism of metal crystal nucleus formation in a single-walled carbon nanotube. *Nat. Chem.* **12**, 921–928 (2020).

11. M. Sleutel, J. Lutsko, A. E. S. Van Driessche, M. A. Durán-Olivencia, D. Maes, Observing classical nucleation theory at work by monitoring phase transitions with molecular precision. *Nat. Comm.* **5**, 5598 (2014)

12. J. Chen, E. Zhu, J. Liu, S. Zhang, Z. Lin, X. Duan, H. Heinz, Y. Huang, J. J. De Yoreo, Building two-dimensional materials one row at a time: Avoiding the nucleation barrier. *Science* **362**, 1135–1139 (2018).

13. M. Ricci, P. Spijker, K. Voïtchovsky, Water-induced correlation between single ions imaged at the solid–liquid interface, *Nat. Commun.* **5**, 4400 (2014).

14. S. H. Loh, S. P. Jarvis, Visualization of ion distribution at the mica-electrolyte interface. *Langmuir* **26**, 9176–9178 (2010).

15. B. A. Legg, M. D. Baer, J. Chun, G. K. Schenter, S. Huang, Y. Zhang, Y. Min, C. J. Mundy, J. J. De Yoreo, Visualization of aluminum ions at the mica water interface links hydrolysis state-to-surface potential and particle adhesion. *J. Am. Chem. Soc.* **142**, 6093–6102 (2020).

16. T. Ando, T. Uchihashi, T. Fukuma, High-speed atomic force microscopy for nano-visualization of dynamic biomolecular processes. *Prog. Surf. Sci.* **83**, 337–437 (2008).

17. K. L. Nagy, R. T. Cygan, J. M. Hanchar, N. C. Sturchio, Gibbsite growth kinetics on gibbsite, kaolinite, and muscovite substrates: Atomic force microscopy evidence for epitaxy and an assessment of reactive surface area. *Geochim. Cosmochim. Acta* **63**, 2337–2351 (1999).

18. S. S. Lee, M. Schmidt, T. T. Fister, K. L. Nagy, N. C. Sturchio, P. Fenter, Structural characterization of aluminum (oxy)hydroxide films at the muscovite (001)−water interface. *Langmuir* **32**, 477–486 (2016)

19. S. A. Saslow Gomez, F. M. Geiger, Precipitates of Al(III), Sc(III), and La(III) at the muscovite−water interface. *J. Phys. Chem. A* **118**, 10974–10981 (2014).

20. J. E. Stubbs, B. A. Legg, S. S. Lee, P. Dera, J. J. De Yoreo, P. Fenter, P. J. Eng, Epitaxial growth of gibbsite sheets on the basal surface of muscovite mica. *J. Phys. Chem. C* **123**, 27615–27627 (2019)

21. G. Sposito, *The Environmental Chemistry of Aluminum* (CRC Press, 1995)

22. B. L. Fulton, C. K. Perkins, R. H. Mansergh, M. A. Jenkins, V. Gouliouk, M. N. Jackson, J. C. Ramos, N. M. Rogovoy, M. T. Gutierrez-Higgins, S. W. Boettcher, J. F. Conley Jr, D. A. Keszler, J. E. Hutchison, D. W. Johnson. Minerals to materials: Bulk synthesis of aqueous aluminum clusters and their use as precursors for metal oxide thin films. *Chem. Mater.* **29**, 7760–7765 (2017).

23. Y.-S. Jun, D. Kim, C. W. Neil. Heterogeneous nucleation and growth of nanoparticles at environmental interfaces. *Acc. Chem. Res.* **49**, 1681–1690 (2016).

24. C. Dai, J. Liu, Y. Hu. Impurity-bearing ferrihydrite nanoparticle precipitation/deposition on quartz and corundum. *Environ. Sci. Nano* **5**, 141–149 (2018).

25. L. M. Hamm, A. J. Giuffre, N. Han, J. Tao, D. Wang, J. J. De Yoreo, P. M. Dove, Reconciling disparate views of template-directed nucleation through measurement of calcite nucleation kinetics and binding energies. *Proc. Natl. Acad. Sci. U.S.A.* **111**, 1304–1309 (2014).

26. Y. Hu, C. Neil, B. Lee, Y.-S. Jun, Control of heterogeneous Fe(III) (Hydr)oxide nucleation and growth by interfacial energies and local saturations. *Environ. Sci. Technol.* **47**, 9198–9206 (2013).

27. D. Turnbull, Kinetics of heterogeneous nucleation, *J. Chem. Phys.* **18**, 198–203 (1950).

28. A. J. Giuffre, L. M. Hamm, N. Han, J. J. De Yoreo, P. M. Dove. Polysaccharide chemistry regulates kinetics of calcite nucleation through competition of interfacial energies. *Proc. Natl. Acad. Sci. U.S.A.* **110**, 9261–9266 (2013).

29. B. A. Legg, J. J. De Yoreo, The energetics of prenucleation clusters in lattice solutions, *J. Chem. Phys.* **145**, 211921 (2016).

30. J. P. Gustafsson, *Visual MINTEQ 3.1 User Guide* (KTH, Department of Land and Water Recources, 2011).

31. A. C. Pan, D. Chandler. Dynamics of nucleation in the ising model. *J. Phys. Chem. B* **108**, 19681–19686 (2004).

32. L. O. Hedges, S. Whitelam. Patterning a surface so as to speed nucleation from solution. *Soft Matter* **8**, 8624–8635 (2012).

33. S. Ryu, W. Cai. Validity of classical nucleation theory for Ising models. *Phys. Rev. E* **81**, 030601 (2010).

34. V. A. Shneidman, R. K. P. Zia Wulff Shapes and the critical nucleus for a triangular Ising lattice. *Phys Rev. B* **63**, 085410 (2001).

35. S. Goldberg, Adsorption models incorporated into chemical equilibrium models, in *Chemical Equilibrium and Reaction Models* (Soil Science Society of America, 1995), pp. 75–95.

36. S. S. Lee, M. Schmidt, N. C. Sturchio, K. L. Nagy, P. Fenter, Effect of pH on the formation of gibbsite-layer films at the muscovite (001)–water interface. *J. Phys. Chem. C* **123**, 6560–6571 (2019).

37. A. Klassen, F. Liu, D. van den Ende, F. Mugele, I. Siretanu. Impact of surface defects on the surface charge of gibbsite nanoparticles. *Nanoscale* **9**, 4721–4729 (2017).

38. T. Hiemstra, "Surface complexation at mineral interfaces: Multisite and charge distribution approach," thesis, Wageningen University, Wageningen NL (2010).

39. A. Imperio, L. Reatto, Microphase separation in two-dimensional systems with competing interactions. *J. Chem. Phys.* **123**, 164712 (2006).

40. M. G. Almarza, J. Pękalski, A. Ciach, Periodic ordering of clusters and stripes in a two-dimensional lattice model. II. Results of Monte Carlo simulation. *J. Chem Phys.* **140**, 164708 (2014).

41. S. M. Loverde, F. J. Solis, M. O. de la Cruz, Charged particles on surfaces: Coexistence of dilute phases and periodic structures at interfaces. *Phys. Rev. Lett.* **98**, 237802 (2007).

42. A Naydenov, P. A. Pincus, S. A. Safran, Equilibrium domains on heterogeneously charged surfaces. *Langmuir* **23**, 12016–12023 (2007).

43. D. Wu, D. Chandler, B. Smit, Electrostatic analogy for surfactant assemblies. *J. Phys. Chem.* **96**, 4077–4083 (1992).

44. D. Andelman, R. E. Rosensweig, Modulated phases: Review and recent results. *J. Phys. Chem. B* **113**, 3785–3798 (2009).

45. T. Smart, H. Lomas, M. Massignani, M. V. Flores-Merino, L. R. Perez, G. Battaglia, Block copolymer nanostructures. *Nano Today* **3**, 38–46. (2008).

46. K. Marstal, F. Berendsen, M. Staring, S. Klein, *SimpleElastix: A User-Friendly, Multi-Lingual Library for Medical Image Registration* (International Workshop on Biomedical Image Registration, 2016).

47. B. A. Legg, M. Zhu, H. Zhang, G. Waychunas, B. Gilbert, J. F. Banfield. A model for nucleation when nuclei are nonstoichiometric: Understanding the precipitation of iron oxyhydroxide nanoparticles. *Cryst. Growth Des.* **16**, 5727–5737 (2016).
